# Clinicopathological correlates in HIV seropositive tuberculosis cases presenting with jaundice after initiating antiretroviral therapy with a structured review of the literature

**DOI:** 10.1186/1471-2334-12-257

**Published:** 2012-10-14

**Authors:** David A Barr, Pravistadevi K Ramdial

**Affiliations:** 1 Empilweni Clinic, Benedictine Hospital, KwaZulu Natal, Nongoma, 3950, South Africa; 2 Brownlee Centre for Infectious Disease, Gartnavel General Hospital, 1053 Great Western Road, Glasgow, G12 0LY, UK; 3 Department of Anatomical Pathology, Level 3, Laboratory Building, Inkosi Albert Luthuli Central Hospital, 800 Bellair Road, KwaZulu Natal, Mayville, 4058, South Africa

**Keywords:** Human immunodeficiency virus, Tuberculosis, Immune reconstitution inflammatory syndrome, Drug induced liver injury, Jaundice, Low resource setting, Liver biopsy

## Abstract

**Background:**

The development of jaundice after initiation of HAART in HIV-TB co-infected patients is a challenging presentation in resource constrained settings, and is often attributed to drug induced liver injury (DILI).Some investigators have described hepatic tuberculosis Immune Reconstitution Inflammatory Syndrome (TB-IRIS) as a cause of liver disease in patients initiating HAART, which could also cause jaundice.

**Case presentations:**

We report the clinical and histopathological features of five HIV-TB co-infected patients presenting with a syndrome of jaundice, tender hepatomegaly, bile canalicular enzyme rise and return of constitutional symptoms within 8 weeks of initiation of highly active antiretroviral therapy (HAART) for advanced HIV infection at a rural clinic in KwaZulu Natal, South Africa.

All five patients had been diagnosed with tuberculosis infection prior to HAART initiation and were on antituberculous medication at time of developing jaundice. There was evidence of multiple aetiologies of liver injury in all patients. However, based on clinical course and pathological findings, predominant hepatic injury was thought to be drug induced in one case and hepatic tuberculosis associated immune reconstitution inflammatory syndrome (TB-IRIS) in the other four.

In these later 4 patients, liver biopsy findings included necrotising and non-necrotising granulomatous inflammation in the lobules and portal tracts. The granulomas demonstrated – in addition to epithelioid histiocytes and Langhans giant cells – neutrophils, plasma cells and large numbers of lymphocytes, which are not features of a conventional untreated tuberculous response.

**Conclusion:**

In this high TB prevalent, low resource setting, TB-IRIS may be an important cause of jaundice post-HAART initiation. Clinicopathological correlation is essential for optimal diagnosis. Further multi-organ based histopathological studies in the context of immune reconstitution would be useful to clinicians in low resource settings dealing with this challenging presentation.

## Background

The roll out of highly active anti-retroviral therapy (HAART) is proceeding rapidly in low and middle income countries, with the number of patients on antiretroviral therapy (ARV) rising from about one quarter million at the end of 2002 to over five million at the end of 2009 [1]. Anecdotally, the development of jaundice after initiation HAART in HIV-TB co-infected patients is more common in low resource, high tuberculosis prevalent areas, and is often attributed to drug induced liver injury (DILI).Some investigators have described hepatic tuberculosis Immune Reconstitution Inflammatory Syndrome (TB-IRIS) as a cause of liver disease in patients initiating HAART, which could also cause jaundice. However, the literature describing this presentation is sparse. We describe five HIV seropositive tuberculosis cases presenting with jaundice after initiating antiretroviral therapy and consider DILI and TB-IRIS as aetiological factors and present detailed clinico-pathological description. This is followed by a structured review of the literature pertinent to these cases.

### Background to cases

This article reports five patients presenting with jaundice within two months of HAART initiation in rural KwaZulu Natal, South Africa and discusses the possible clinicopathological causes with a focus on tuberculosis-associated immune reconstitution inflammatory syndrome (TB-IRIS) involving the liver. All five cases were originally included in a prospective audit of HAART initiation in a rural district level ARV clinic in Nongoma, KwaZulu Natal, South Africa. This audit recorded baseline and follow-up data at 2 and 8 weeks post-HAART initiation for 90 consecutive adult patients referred for HAART initiation between 18^th^ June and 27^th^ July 2010. The population that the cases are drawn from can therefore be accurately described from the audit sample (Table 1).

**Table 1:** Baseline characteristics of patient population case series is drawn from

**Baseline characteristic**	**Value**
Median age in years (IQR)	34 (29 to 40)
Median CD4 cells/μL (IQR)	135 (70 to 194)
%female	67%
% with cultured confirmed TB as part of HAART initiation work up	~40%
% diagnosed with TB as part of HAART initiation work up	~50%
% with alanine transaminase elevation grade ≥ 1	28%
% with alanine transaminase elevation grade ≥ 3	3%
% with alkaline phospatase > 98U/L (female) or > 128U/L (male) at time of HAART initiation	43%

IQR = Inter-quartile range.

Of the 90 patients in this audit series, six presented with jaundice during 8 weeks of follow up post-HAART initiation. Five of these six gave written consent to have their cases reported and are presented below, the first and fifth cases are described in detail while the remaining three are summarised. All five patients underwent liver biopsy and Table 2 details the microscopic findings.

**Table 2:** Summary of histopathological findings

**Feature**	**Patient 1**	**Patient 2**	**Patient 3**	**Patient 4**	**Patient 5**
**Hepatocytes**					
Swelling	mild	marked	mild	mild	marked
Regeneration	Present	Present	Present	Present	Present
Steatosis	Patchy	Diffuse	Patchy	Patchy	Diffuse
Cholestasis	Present	Present	Present	Present	Present
**Inflammation**					
NGI	Portal/parenchymal	Portal/parenchymal	Portal/parenchymal	Absent	Absent
NNGI	Portal/parenchymal	Portal/parenchymal	Portal/parenchymal	Absent	Absent
Langhans GC	NGI/NNGI	NGI/NNGI	NGI/NNGI	Absent	Absent
NPs	NGI	NGI & parenchyma	NGI	parenchyma	*portal tracts & parenchyma
LCs	Portal/parenchymal; T dominant (admixed CD4^+^/CD8^+^)	Portal/parenchymal; T dominant (admixed CD4^+^/CD8^+^)	Portal/parenchymal; T dominant (admixed CD4^+^/CD8^+^)	nil	Portal/parenchymal; T dominant (dominant CD8^+^)
PCs	Portal	Portal	Portal & parenchymal	nil	Portal & parenchymal
EPs	Portal/parenchymal	Portal/parenchymal	Portal/parenchymal	nil	*Portal/parenchymal
HCs	Portal/parenchymal	Portal/parenchymal	Portal/parenchymal	nil	Portal/parenchymal
**Other**					
Architecture	Maintained	Mixed cirrhosis	Maintained	Cannot comment	Maintained
Fibrosis	Portal	Fibrous linkages	Portal	Cannot comment	nil
I/hepatitis	Absent	Absent	Absent	Cannot comment	Present
ZN	Paucibacillary	Negative	Negative	Not done- no tissue	Negative
PCR	Positive	Positive	Positive	Not done – no tissue	Negative
Haemosiderin	Present	Present	Present	Present	Present

Key: EPs: presence and dominant location of eosinophils; HC: presence and dominant location of histiocytes; I/hepatitis: interface hepatitis; Langhans GC: presence and location of Langhans giant cells; LCs: presence, dominant location & subtype of lymphocytes; NNGI: presence and location of non-necrotising granulomatous inflammation; NGI: presence and location of necrotising granulomatous inflammation; NPs: presence and dominant location of neutrophils; *PCs* presence and dominant location of eosinophils; *PCR* polymerase chain reaction; *ZN* ZiehlNeelsen stain.

## Case presentations

### Patient 1

A 58 year old woman with a CD4 count of 23cells/μL was referred for HAART initiation by a local general practitioner. She had a 2 week history of fever, night sweats, increasing dyspnoea and right sided chest pain, in addition to 7 days of lethargy and fast palpitations. She could walk only with assistance of 2 people. A large right sided pleural effusion was detected, which was confirmed to be a lymphocytic exudate on cytological and chemical testing. Apart from a mildly raised alkaline phosphatase, liver function tests (LFTs) were normal, and hepatitis B surface antigen was negative. She was admitted for inpatient care and commenced on empirical quadruple anti-tubercular therapy as per South African national guidelines [2] (rifampicin, isoniazid, pyrazinamide and ethambutol), along with a 5 day course of ceftriaxone and prophylactic co-trimoxazole. In addition 4 litres of fluid was drained from the pleural space. Induced sputum and pleural fluid were acid fast bacilli (AFB) negative.

After two weeks, the fever, night sweats, chest pain and palpitations had resolved. She could walk unaided. A small pleural effusion and mild abdominal tenderness in the right upper quadrant remained, but there was no hepatomegaly. She was commenced on efavirenz, tenofovir and lamivudine as per national guidelines [3].

**Figure 1:**
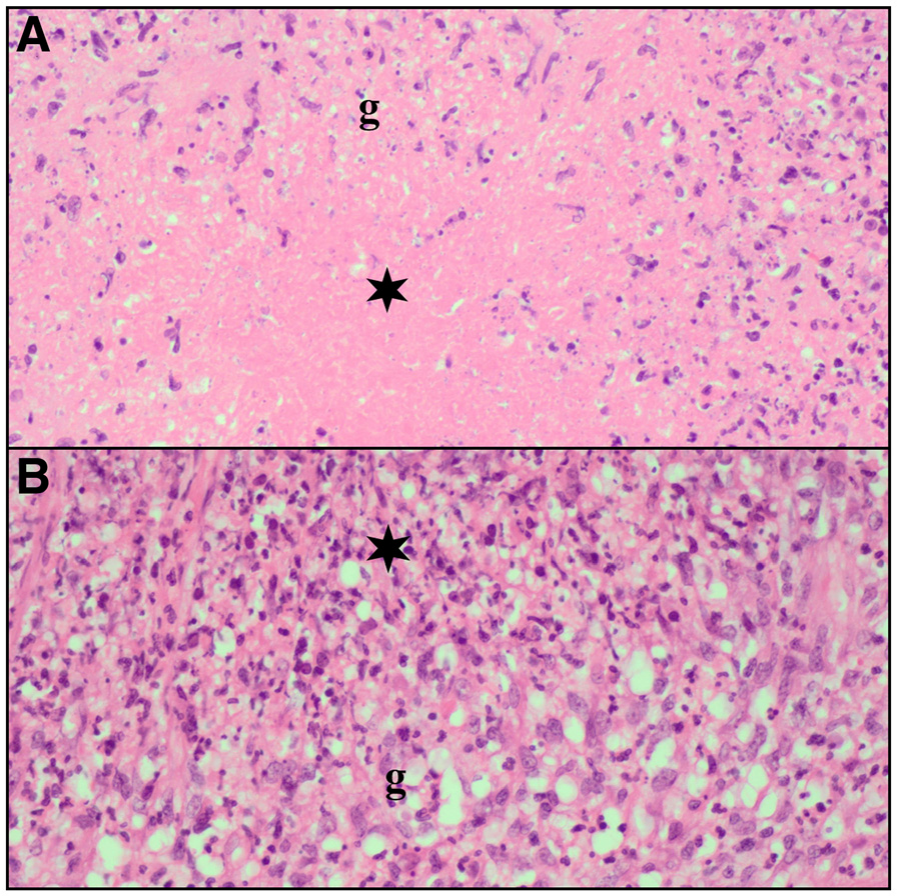
**(Patient 1) Necrotising granulomatous inflammation (g) with mixed inflammatory cells and caseative necrosis (A, asterisk) and suppurative inflammation (B, asterisk) *[haematoxylin & eosin].***

**Figure 2:**
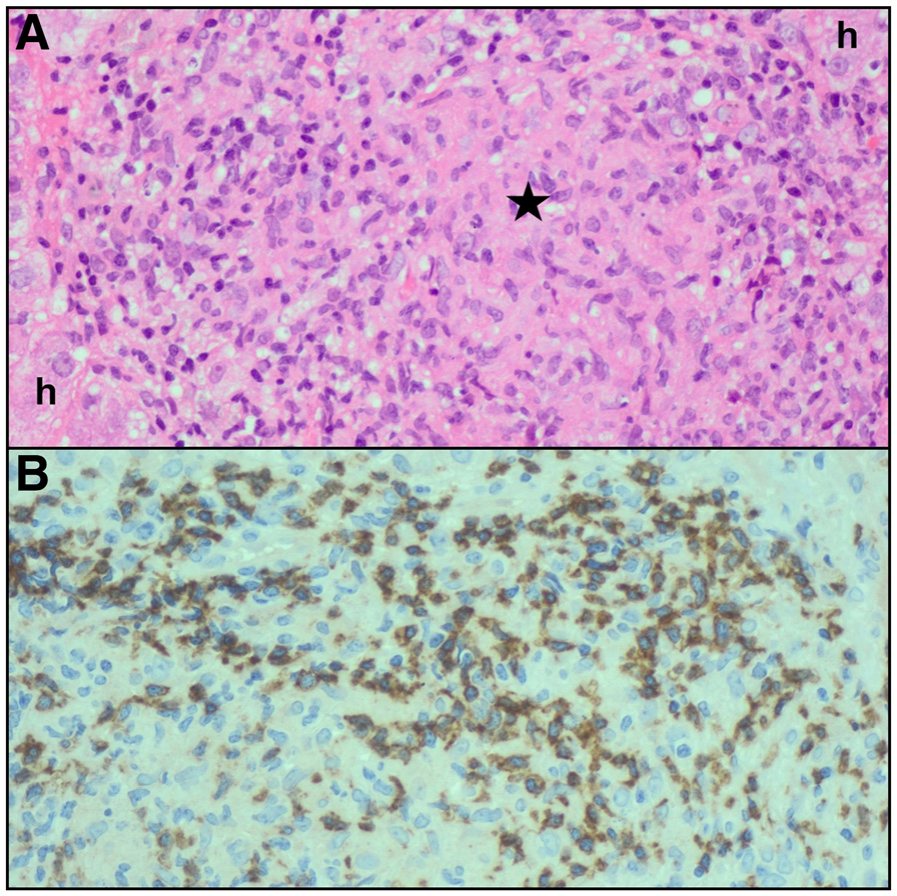
**(Patient 1) Lobular non-necrotising granulomatous inflammation (A) with central aggregation of epithelioid histiocytes (asterisk) (h = hepatocytes) *[hematoxylin & eosin] *, and prominent CD8+ T-lymphocytic infiltrate (B).**

At routine review two weeks post-HAART initiation she was found to be confused with fever, dyspnoea, and chest pain. She had again lost her ability to walk unaided. Examination revealed jaundice, a re-accumulated pleural effusion and new tender hepatomegaly. Ultrasound scan of the abdomen identified epigastric lymph nodes >3 cm in diameter and an enlarged liver with abnormal texture. The LFTs demonstrated marked elevation in bile canalicular hepatic enzymes; raised bilirubin was predominantly conjugated, and clotting studies were normal (Table 3). A liver biopsy was performed which showed necrotising (Figure 1A, 1B) and non-necrotising (Figure 2A) granulomatous inflammation with AFB. Some of the necrotising granulomas demonstrated caseative necrosis (Figure 1A) while others were suppurative (Figure 1B). All the granulomas also contained a lymphocytic infiltrate with an admixed CD3^+^/CD4^+^ and CD3^+^/CD8^+^immunoprofile. The latter predominated (Figure 2B). There was also cholestasis and a mixed, predominantly macrovesicular,steatosis, and no viral inclusions (Table 2). Pleural fluid and mycobacterial blood culture sent at the time of first presentation confirmed fully sensitive *Mycobacterium tuberculosis*.

**Table 3:** Chronological selected blood indices for patient 1 and 5

**Week since starting HAART**		**Alb**	**Total Bil**	**ALT**	**ALP**	**GGT**	**Hb**	**WCC**	**plt**	**lactate**	**INR**
**Patient 1**											
−2	First visit to ARV clinic	23	5	13	152	73	7.4	6.5	394	n/a	
0		26	11	20	131	89	8.0	5.5	314	n/a	
+2	Presents with jaundice 2 weeks post HAART	20	88*	127	833	429	7.0	3.1	803	2.9	1.3
+4	Starts 14 days prednisolone	17	62	99	1310	673	7.0	2.9	736	n/a	1.0
+8	Clinically improved	24	22	49	667	330	7.9	3.9	448	n/a	1.0
**Patient 5**											
−2	First visit to ARV clinic	22	18	34	119	35	11.3	5.4	364	n/a	n/a
0	After 2 weeks TB treatment starts HAART	24	21	45	133	57	9.3	4.4	682	n/a	n/a
+2	Has been found to have jaundice 10 days post HAART; TB treatment stopped	21	56*	60	115	99	n/a	n/a	n/a	2.7	1.3
+3	With resolving jaundice and clinical deterioration TB treatment restarted	26	26	48	138	93	9.5	2.6	460	2.6	1.2
+4	Return of jaundice and liver biopsy suggests DILI – TB treatment stopped	21	97	67	187	148	9.2	2.7	424	1.6	1.4

* > 85% conjugated bilirubin.

*Alb* albumin in g/L, *Bil total* total bilirubin μmol/L, *ALT* alanine aminotransteraseiu/L, *ALP* alkaline phosphatase iu/L, *GGT* Gamma-glutamyltranspeptidaseiu/L., *Hb* haemoglobin in g/dL; WCC = white cell count (total) x10^9^/L; plt = platelets x10^9^/L; lactate is measured in mmol/L; INR = international normalised ratio.

The jaundice was felt to be primarily due to hepatic TB IRIS. HAART and TB therapy were continued, and a 14 day course of prednisolone 30 mg OD prescribed. All symptoms resolved over a 4 week period, along with the normalisation of observation chart variables, resolution of the hepatomegaly and pleural effusion.

### Patients 2, 3 and 4

The clinical and laboratory features of patients 2, 3 and 4 were similar to patient 1, and are summarised below. TB-IRIS was favoured as a primary cause of jaundice in these three cases. The histopathological features of patients 2 and 3 were also similar to patient 1. The biopsy from patient 4 was inadequate for optimal appraisal and investigation; this patient’s working diagnosis was based on clinical and non-biopsy laboratory findings.

Patient 2 was a 33 year old man with a history of problem alcohol use and pleural TB treated the previous year. He had a CD4 count of 61 cells/μL and had repeat treatment for pleural and pericardial TB starting three weeks prior to HAART initiation. After four weeks of HAART he deteriorated with re-accumulated effusions, plus new jaundice and tender hepatomegaly. Liver enzymes were cholestatic and abdominal ultrasound showed diffusely abnormal liver with epigastric lymphadenopathy. He was thought to have underlying chronic liver disease secondary to hepatitis B virus and alcohol consumption, with a superadded hepatic TB-IRIS, and was successfully managed with continued HAART and TB drugs with inpatient monitoring of liver enzymes.

Patient 3 was a 38 year old female diagnosed commenced on pulmonary TB therapy 2 weeks prior to HAART initiation (CD4 32 cells/μL). She then did not attend for follow up for 6 weeks, when she had deteriorated with systemic symptoms and functional decline, plus a worsened chest x-ray. Jaundice and tender hepatomegaly were noted, with cholestatic pattern liver enzymes. Disseminated TB-IRIS was suspected (including hepatic); TB therapy and HAART were continued but prednisolone not given as sensitivity results from mycobacterial culture were still pending. She made a full recovery.

Patient 4 was a 41 year old man with CD4 123 cells/μL who had a prolonged hospital admission during which he was treated for disseminated tuberculosis infection, being commenced on HAART on discharge (after 4 weeks TB therapy). At two week review he was unwell with systemic symptoms, jaundice, new hepato-splenomegaly, epigastric lymphadenopathy and a deteriorated chest x-ray. Although ALT was significantly raised (grade 3) liver enzyme derangement was mixed, predominantly cholestatic. A fully sensitive Mycobacterium tuberculosis was grown from his induced sputum and he was treated for multi-systemic (and hepatic) TB-IRIS with prednisolone and made a rapid recovery.

Additional clinical details on cases 2,3 and 4 are available in Additional file 1: Table S1.

### Patient 5

A 34 year old woman with CD4 count 17 cells/μL was referred from a local hospital outpatient department for initiation of HAART. However, she reported 2 months intermittent diarrhoea which was watery with associated abdominal cramp pain, and vomiting after food. There was also a 2 month history of lethargy, anorexia, and weight loss. On direct questioning she also described a dry cough for the last 4 days. She had been diagnosed previously with tuberculosis, and finished treatment 4 months prior to this presentation.

Examination findings included cachexia, tachycardia, temperature of 37.7°C, oral candidiasis and a long standing hyperpigmented rash of prior pruritic papular eruption. Abdominal palpation revealed a tender epigastric fullness and possible mass or medial liver enlargement. Chest radiology demonstrated calcified hilar nodes and a sub-pulmonic effusion while ultrasound scanning confirmed pleural fluid and epigastric lymphnodes greater than 3 cm and hepatomegaly of normal texture. Routine blood biochemistry and full blood count results included mild hyponatraemia, normocytic anaemia, and hypoalbuminaemia. Induced sputum was sent for mycobacterial culture, and stool and blood were sent for bacterial culture, but the pleural effusion was too small for aspiration. She was commenced on quadruple therapy for tuberculosis plus streptomycin as a ‘re-treatment’ case of pleural tuberculosis as per the national South African guidelines. Prophylactic dose co-trimoxazole was also initiated.

Two weeks later the pyrexia had resolved, but the diarrhoea and tachycardia persisted and she had a postural drop in blood pressure. Admission for intravenous fluids and antibiotics for gastroenteritis (ceftriaxone, erythromycin) was arranged and HAART was initiated (efavarenz, lamivudine and tenofovir as per national guidelines). Previous aerobic blood cultures and stool culture showed no growth.

The vomiting and diarrhoea had resolved 10 days into admission but the patient was noted to have jaundice, high fever, on-going tachycardia and weight loss. On examination there was now a 4 finger width tender hepatomegaly and a moderate sized right sided pleural effusion. The ward doctor discontinued tuberculosis treatment and continued co-trimoxazole and HAART.

Over the next 7 days there was continued fever and weight loss, with some resolution of abnormal liver function tests (Table 3). Aspirate of the pleural effusion confirmed a lymphocytic exudate. Ultrasound imaging showed an enlarged liver, normal biliary tree, normal spleen, no large nodes, and no free fluid; a liver biopsy was performed.

**Figure 3:**
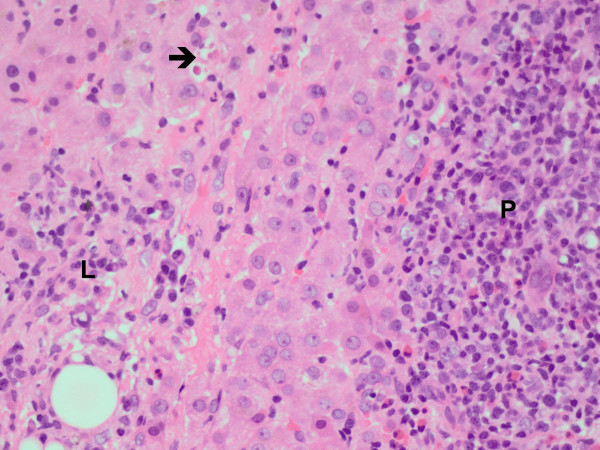
**(Patient 5) Dense, predominantly lymphoplasmacytic, portal (P) inflammatory infiltrate with scattered eosinophils and lobular necroinflammatory foci (L, arrow) with apoptotic hepatocytes (arrow) *[hematoxylin & eosin].***

After a 10 day interruption, because of improved liver biochemistry and progressing clinical deterioration felt to be due to disseminated tuberculosis, full TB treatment was restarted. This again resulted in worsening liver biochemistry (Table 3). The liver biopsy (Table 2)(Figure 3) demonstrated necroinflammatory foci with eosinophils, marked hepatocyte swelling, regenerative activity with multinucleation and diffuse mixed macro-and-microvesicularsteatosis and cholestasis. The portal tracts were expanded by a mixed, but predominantly lymphoplasmacytic and eosinophilic, infiltrate. There was irregularity of the portal-parenchymal interface in 2 portal tracts with conspicuous destruction of hepatocytes by lymphocytes in these foci. Granulomas were not identified. Mycobacterial PCR investigation was negative. Tuberculosis therapy was stopped for a second time with a plan to reintroduce at a later date using a drug hepatotoxicity protocol. However, all mycobacterial culture results (induced sputum and pleural fluid) were negative at 42 days, the patient’s symptoms resolved and she gained weight. It was felt her jaundice was primarily drug induced liver injury, although cholestasis relating to bacterial sepsis is also a possibility. The lymphocytic pleural exudate and abdominal lymphadenopathy may have been a paradoxical reaction to residual TB antigen from her previously treated tuberculosis. She remained well without TB treatment at 12 months follow up.

## Discussion

All five patients discussed above had a diagnosis of tuberculosis made pre-HAART and were on TB therapy at time of HAART initiation. They were immunosuppressed, as evidenced by clinical findings and CD4 count. All presented with new tender hepatomegaly, jaundice with bile canalicular enzyme rise and preserved liver synthetic function, as evidenced by normal clotting studies and return of constitutional symptoms within 8 weeks of initiation of HAART. Ultrasound scan was not diagnostic in any of the patients; in particular none had bile duct dilatation. There was evidence of multiple potential aetiologies in all patients, but the predominant cause was felt to be DILI in one case and hepatic TB- IRIS in the other four. We discuss these findings in the context of previous literature under the headings liver disease in HIV infection, drug induced liver injury and hepatic TB IRIS below.

### Liver disease in HIV infection

In the pre-HAART era abnormal liver enzymes were regarded common in HIV, with prevalence rising with advancing immunosuppression [4,5]. Histopathology studies demonstrated a multiplicity of HIV associated disease in the liver, with aetiology differing widely in different population settings [6-10]. Despite this, jaundice has been regarded as infrequent in HIV [11]. Again, as might be expected, the incidence and aetiology of jaundice in HIV-infected patients may vary in different populations [12,13].

Since the introduction of HAART there has understandably been greater focus on drug associated hepatotoxicity in HIV. In high income settings, chronic liver disease – associated with Hepatitis C and B virus, long term drug-induced toxicity, alcohol related and non-alcoholic fatty liver disease – has become a leading cause of chronic morbidity and mortality in people living with HIV [14]. Mechanisms underlying long-term hepatic injury are probably distinct from hepatotoxicity seen acutely after introduction of HAART [15]. Early severe hepatotoxicity (SH) is often arbitrarily defined as elevated serum aminotransferases >5 times the upper limit of normal (grade 3 and above by the AIDS Clinical Trial Group classification) within the first 6 months of HAART; in randomised controlled trials (RCTs) incidence of SH ranges from 2 to 18% [16], and may have even greater variability in observational studies [17].

However there are well described problems with assessing hepatotoxicity of HAART in randomised and observational trials [18]. Asymptomatic elevations in liver enzymes may not be an accurate surrogate for risk of developing rare, clinically relevant, liver disease. Furthermore, the biochemical definition of hepatotoxicity is not standardised despite use of the AIDS Clinical trial Group (ACTG) classification. Although ACTG give parameters for significant elevation of all liver enzymes [19], many studies only include transaminase elevations – raised total bilirubin is often inconsistently defined, and alkaline phosphatase rarely considered. Which liver enzymes are included can have a large effect on conclusions [20]. In addition, when considering patients with *baseline* abnormalities of transaminases investigators generally use modified ACTG criteria (for example elevations of transaminases > 3.5 times baseline), but such modifications are not standardised. Finally, upsets in liver enzymes can occur in the absence of HAART, but in hepatotoxicity studies all significant hepatic abnormalities are attributed to “toxicity”, or it is often not clear how investigators have excluded cases due to other causes.

Nevertheless, it is clear that, again, in high income settings co-infection with viral hepatitides is pivotal in determining risk of early hepatotoxicity [21-24]. By comparison, HIV cohorts in limited resource settings tend to have much lower rates of Hepatitis C infection, and are more likely to use nevirapine in first line regimens. Hepatotoxicity studies in these settings reveal a heterogeneous picture. Reported rates of early hepatotoxicity in people initiating HAART in African studies are generally low at 1-2% [25-33] but occasionally much higher at 3.4-18.4% [34-37]. Grade 1 or 2 rises in liver enzymes are very prevalent in all these studies. Some well conducted studies have reported a significantly higher rate of early grade 3 or 4 hepatotoxicity in patients treated with nevirapine compared to efavarenz [25,37], while other studies have not [26]. Co-infection with viral hepatitides is not found to be a significant risk factor for hepatotoxicity [25,26,28,34,37], in most but not all studies [35]. In contrast, co-treatment with anti-tuberculous medications is found to be a major risk factor in African cohorts [25,28,35], although this also has exceptions [29].

Different follow up times and frequencies of serum liver enzyme assessment may explain much of this variation – for example the studies which have found high rates of early nevirapine associated hepatotoxicity have higher rates of blood sampling in the first 3 months post-HAART initiation, which is when most hepatotoxicity was identified, suggesting other studies may simply have missed this occurrence.

In correspondence with the high income setting studies, there is also the problem of definition of hepatotoxicity. This difficulty is underlined in light of the finding that point prevalence of ‘grade 3 or 4 hepatotoxicity’ may be very similar before and after HAART initiation [35], or that HAART initiation even reduces the rates of transaminitis in African HIV treatment cohorts [28]. This suggests trials which define HAART related hepatotoxicity as any significant rise in liver enzymes after HAART initiation have oversimplified a complex, multifactorial problem.

Helping get beyond these problems are papers that report clinically relevant outcomes, which several studies in an African setting do. Reported rate of changing HAART regimen in response to hepatotoxicity is gene rally lower than reported rate of grade 3 or 4 liver enzyme rise – for example 0.7% v. 1.4%; [30] 9.1% v. 13.8%;[37]; 2.6% v. 3.4%; [36] 0.9% v. 4.6% [35]. It is possible that clinicians do not always think such rises are heralding life threatening drug induced liver injury, perhaps relying instead on additional features such as lactic acidosis, hypersensitivity rash or eosinophilia or are reassured by the transient nature of a rise. Several investigators have recorded that grade 3 or 4 liver enzyme abnormalities often resolve spontaneously without change in medications [29,37]. However, some investigators have noted higher incidence of symptoms in those with compared to those without grade 3 or 4 liver enzyme elevation [35,37]. Importantly, while deaths attributed to HAART hepatotoxicity are infrequent, they do occur; reported rates are 0 to 0.5% [35-37].

Further clinical context is provided in some hepatotoxicity studies. In reports of hepatotoxicity in a treatment cohort in rural Uganda [28,38], Weidle and colleagues describe clinical hepatitis – defined as presence of jaundice, liver enlargement and gastrointestinal symptoms – in 4 out of 1029 enrolled patients within 3 months of HAART initiation. Although 12.7% of this cohort received TB treatment, none of the 4 patients with clinical hepatitis were on anti-mycobacterial medication at the time of their reactions. All 4 patients also exhibited a hypersensitivity rash, one case was caused by co-trimoxazole, and 3 were attributed to nevirapine; one of the 4 patients died from the reaction.

Kalyesubula et al. reporting from urban clinics in Kampala give a detailed account of clinical and biochemical surveillance for hepatitis in HIV seropositive patients [25]. In a cohort of 236 individuals, 66 developed new transaminitis within 14 weeks of commencing HAART, although only 3/66 were grade 3 or 4 rises. The investigators report that, in the 66 patients with any aminotransferase elevation, 33% had vomiting, 20% right upper quadrant pain, 17% hepatomegaly and 8% jaundice. Although only 8/236 patients were receiving any TB treatment, the authors found current TB therapy to be a risk factor for any grade 2 – 4 transaminitis, but it is not clear what clinical findings were observed in HIV-TB co-infected patients who developed transaminitis.

Unlike in this previous work, the current case series is defined by a clinical presentation rather than the presence or absence liver enzyme elevation. In addition, we present a detailed clinico-pathological description in an attempt to better define the causes of the liver disease in these patients, which is not found in the literature on hepatotoxicity post-HAART. It should be emphasised that the treatment cohort these cases are drawn from may be quite atypical compared to some of the above studies. In particular, the very high rate of culture confirmed tuberculosis in our cohort (about 40% from samples obtained during HAART initiation work up).

### Drug Induced Liver Injury (DILI)

There is no gold standard for the diagnosis of DILI. The clinical and histopathological spectrum of DILI is wide and overlaps with other hepatic diseases. The more common hepatitic pattern of DILI, with ALT >5 times upper limit of normal, was not apparent in these five patients who had predominant bile canalicular enzyme elevation. However, cholestatic or mixed hepatocellular/cholestatic DILI has been reported with use of rifampicin, [39] isoniazid [40], ethambutol [41], and co-trimoxazole [42], hence, drug hepatotoxicity must still be considered.

In lieu of a gold standard diagnostic test, clinical scales for diagnosis of DILI have been developed. One of the most validated is the Maria &Victorino (M&V) system [43]. Based on the M&V system score, patient 5 above might be considered a ‘possible’ DILI, while patients 1 to 4 score as ‘unlikely’ to be DILI. Caution must be taken in application of DILI clinical scales: poor reproducibility along with inter-rater and inter-scale disagreement have been shown [44], and they are not validated in HIV- positive populations or low resource settings. Despite these limitations, from a clinical perspective, worsening of LFTs on drug re-challenge in patient 5 is strong evidence of DILI, and normalisation of LFTs without interrupting therapy is strong evidence that DILI was not a predominant cause of the jaundice seen in the four other cases.

Histopathologic assessment of possible DILI can also be problematic. Drug hepatotoxicity can produce a spectrum of acute hepatic pathology that imitates a range of patterns described in primary hepatic disease [45,46]. This mimicry poses a major diagnostic challenge because the shared histopathological features, comprising mainly necro-inflammatory hepatocellular, cholestatic and mixed hepatocellular-cholestatic patterns, cannot be attributed unequivocally to drugs [45,47]. Although this wide spectrum of histopathological reaction patterns displayed in DILI includes a granulomatous reaction pattern, drug-induced granulomatous responses are usually non-necrotising. Because 3/5 patients in the present study demonstrated necrotizing granulomatous inflammation, a drug-induced cause is morphologically inappropriate. This is also strengthened by the identification of acid fast bacilli in one biopsy and confirmation of an *M. tuberculosis complex* footprint by polymerase chain reaction.

One of the five biopsy results in these cases was considered strongly suggestive of DILI and resulted in decision to interrupt TB medications (case 5, Figure 3, histology showed moderate portal tract and lobular inflammation with eosinophils and interface hepatitis). The presence of eosinophils is a well-recognised feature of drug reactions in many organs, but caution is necessary in the interpretation of their presence. This is particularly pertinent in the current context, because patients with advanced AIDS demonstrate a dominant Th2 response with associated tissue “eosinophilia”. Furthermore, silent parasitic infections may be responsible for heightened eosinophil numbers. Notwithstanding this, however, whilst all biopsies in the present study contained eosinophils, the density of eosinophils was most intense in the biopsy from patient 5. In this biopsy, there were no other histopathological clues of a parasitic infestation.

In summary, no gold-standard exists for diagnosis of DILI, but liver biopsy with clinicopathological correlation suggests DILI as a probable primary cause of hepatic disease in only one of the five cases presented here.

### Hepatic TB-IRIS

Although tuberculosis of the liver is not commonly diagnosed clinically, it is a frequent post-mortem finding in HIV positive patients [48]. It is possible that hepatic tuberculosis (IRIS) may be similarly under recognised. Lawn and Wood [49] report not infrequent involvement of the liver in South African patients with TB-IRIS – four patients in a case series of seventeen TB-IRIS patients. They suggest that predominant rise in bile canalicular enzymes, liver capsular pain, manifestations of TB-IRIS at another anatomical site, are all suggestive features, while jaundice may develop in some patients.

Meintjes et al. [50] have also shown high prevalence of cholestatic pattern liver enzyme derangement and hepatomegaly in South African patients diagnosed with TB-IRIS in a large prospective observational study, but do not comment specifically on jaundice. Similarly, Haddow et al. [51] report a high frequency of elevated liver enzymes in a cohort of South African patients initiating HAART who were consequently classified as possible cases of hepatic TB-IRIS by expert consensus. By contrast other TB-IRIS cohort studies do not report any liver involvement [52]. Such variability may represent differences in study populations or may relate to diagnostic difficulty. In likeness with DILI, TB-IRIS has no diagnostic gold standard test, but a consensus clinical case definition has been proposed for low income settings [53]. Four of the five jaundiced patients in this series were felt to have met this TB-IRIS case definition (Additional file 1: Table S2).

A specific case definition for hepatic TB-IRIS has also been suggested, which additionally requires the presence of granulomatous inflammation on liver biopsy [50]. Lawn and Wood suggest that large epithelioid granulomatous inflammation seen on liver biopsy is evidence of hepatic TB-IRIS [49]. In the present study, not only was necrotising and non-necrotising granulomatous inflammation present in the lobules and portal tracts, but the granulomas demonstrated – in addition to epithelioid histiocytes and Langhans giant cells – neutrophils, plasma cells and large numbers of lymphocytes, which are not features of a conventional untreated tuberculous response. The microscopic spectrum of the tuberculous IRIS reaction is poorly documented, but a similar dense neutrophilic and lymphoplasmacytic inflammatory response has been documented in reaction to cryptococcal antigen in tissue [54]. Although the present study is limited by the absence of interval CD4 counts and viral load measurements, in view of the other laboratory findings and clinical history, including patient outcome, the spectrum of histopathological findings is proposed as the expanding morphological profile of tuberculous granulomas in the setting of IRIS. We believe this is the most detailed clinico-pathological description of hepatic TB-IRIS to date. Further multi-organ based histopathological studies in multiple organs are necessary for such validation.

### Multiple causes of jaundice in individual patient

Immune-compromised patients are often found to have multiple concurrent liver pathologies [55]. The majority of cases presented above have evidence of more than one potential cause of jaundice. A further contributor to jaundice in these cases could be the isolated hyperbilirubinaemia (without evidence of cholestasis or hepatocellular damage) related to rifampicin interference with bilirubin uptake [56-58]. Heller et al. [59] postulate this may be at least a partial cause of their ‘relatively frequent’ observation of jaundice shortly after initiation of TB medications in South African patients. This might be particularly relevant to patient 5 in this case series, who had jaundice in the presence of only minor alkaline phosphatase elevation.

### Preliminary guidelines on how healthcare workers in resource-limited settings should respond to jaundice in HIV positive TB cases on antiretroviral therapy

A lack of evidence exists informing the management of the clinical presentation of jaundice in this context and the preliminary nature of the following guidance should be emphasised.

● Presentation with jaundice in HIV positive patients on TB treatment and HAART should prompt full clinical assessment. Bloods for biochemical and haematology assays, and ultrasound assessment of the abdomen should be performed if available.

● Elevations in transaminases or bilirubin >5x upper limit of normal, or >3.5x the baseline level, are not typical of hepatic TB-IRIS. If present, clinicians should strongly suspect DILI and follow local or national guidelines for hepatotoxicity from anti-tuberculosis and anti-retroviral medications.

● The presence of hypersensitivity rash, lactic acidosis, significant coagulopathy or eosinophilia is strongly suggestive of DILI and clinicians should follow local or national guidelines for hepatotoxicity from anti-tuberculosis and anti-retroviral medications.

● In cases where anti-tuberculosis and/or anti-retroviral medications have been discontinued but liver enzymes are failing to resolve, liver biopsy should be considered if available.

● Features suggestive of hepatic TB-IRIS as a cause of jaundice include:

 ● evidence of IRIS in another system;

 ● new tender hepatomegaly;

 ● predominantly cholestatic liver test derangement;

 ● abdominal ultrasound findings of diffusely abnormal liver texture, lymphadenopathy and normal biliary tree.

● If diagnostic uncertainty exists clinicians should consider liver biopsy, if available, or transfer to a higher level of care.

● Histopathological findings which suggest TB-IRIS in liver tissue include large epithelioid granulomatous inflammation. Features which are atypical of conventional tuberculous response – including neutrophils, plasma cells and large numbers of lymphocytes – may also be present.

● Multiple causes of liver disease are common in HIV-TB co-infected patients presenting with jaundice following HAART initiation, and additional diagnoses such as bacterial sepsis, DILI from traditional medicines, and active hepatitis B infection should be actively sought.

● Causes of liver disease in HIV-TB co-infected patients show wide variation in relative frequency according to geographical location and population studied; clinicians should be aware of local and national experience for their setting.

## Conclusion

HIV seropositive tuberculosis cases presenting with jaundice after initiating antiretroviral therapy represent a challenge to clinicians working in resource constrained settings. We believe hepatic TB-IRIS is an important aetiological factor in our setting. Fastidious clinicopathological correlation is essential for optimal diagnosis. Prospective histopathological studies in multiple organs for better characterisation of the morphological profile of tuberculous granulomas in the setting of immune reconstitution are necessary to aid clinical decision making in difficult cases such as those presented here.

## Consent statement

Written informed consent for publication was obtained from the 5 patients whose cases are presented above. A copy of the written consent is available for review by the Series Editor of this journal.

## Competing interests

The authors declare that they have no competing interests.

## Authors’ contributions

PKR performed histopathological assessments; clinical assesments and details were provided by DAB. Both authors drafted the manuscript. All authors read and approved the final manuscript.

## Pre-publication history

The pre-publication history for this paper can be accessed here:

http://www.biomedcentral.com/1471-2334/12/257/prepub

## Supplementary Material

Additional file 1 **Table S1.** Summary of clinical details for cases 2 to 5. Clinical details are given at 4 chronological stages: initial review, review at time of HAART initiation, presentation with jaundice post-HAART initiation, and outcome. **Table S2.** Application of Meintjes et al Paradoxical TB Case Definition to cases. Click here for file
