# Supplementary material for: Clinicopathological correlates in HIV seropositive tuberculosis cases presenting with jaundice after initiating antiretroviral therapy with a structured review of the literature
Source: BMC Infect Dis. 2012 Oct 14;12:257. doi: 10.1186/1471-2334-12-257 (PMC3526386; doi:10.1186/1471-2334-12-257)
Supplement: Additional file 1 — Table S1. Summary of clinical details for cases 2 to 5. Clinical details are given at 4 chronological stages: initial review, review at time of HAART initiation, presentation with jaundice post-HAART initiation, and outcome. Table S2. Application of Meintjes et al Paradoxical TB Case Definition to cases. [file 1471-2334-12-257-S1.docx]

# Supplemental tables

**Table S1: Summary of clinical details for cases 2 to 5.** Clinical details are given at 4 chronological stages: initial review, review at time of HAART initiation, presentation with jaundice post-HAART initiation, and outcome.

| **Initial review at ARV clinic and TB diagnosis** | | **Review at time of HAART initiation** | **Clinical course after starting HAART** | **Clinical outcome** |
| --- | --- | --- | --- | --- |
| **Patient 2: 33 year old male; CD4 61 cells/µL** | | | | |
| - Prior history of pleural TB 18months ago, and heavy alcohol use. - Examination and USS reveal 3cm pericardial effusion, massive right pleural effusion (lymphocytic exudate), normal liver, epigastricadenopathy. - Commenced on RHZES, pyridoxine, prophylactic CTX. | | After 3 weeks RHZE denies all symptoms. Right pleural effusion now smaller, heart sounds normal, abdomen non-tender. Commenced on HAART. | - Remained well at 2 week review post-HAART initiation - At 4 week review noted to have return of symptoms, jaundice, re-accumulated pleural effusion, tender hepatomegaly. - USS: abnormal texture enlarged liver, epigastric lymph nodes now >3cm. | The aetiology of the jaundice was thought to be multifactorial, with hepatitis B infection, alcoholic liver disease, hepatic TB and drug toxicity all possible.  HAART and TB drugs continued with inpatient monitoring LFTs. Over 4 weeks jaundice, fever, tender hepatomegaly resolved and gained weight. |
|  | LFTs at time of initial presentation: | LFTs at time of HAART initiation: | LFTs at time of presentation with jaundice: | LFTs 6 weeks after presentation with jaundice: |
| Alb | 19 | 24 | 19 | 26 |
| Bil total | 24 | 27 | 55 | 28 |
| ALT | 40 | 33 | 61 | 44 |
| ALP | 120 | 109 | 113 | 98 |
| GGT | 53 | 73 | 74 | 45 |
| **Patient 3: 38 year old female; CD4 32 cells/µL** | | | | |
| - Reports 4 weeks dry cough, weight loss, night sweats and anorexia; found to be pyrexial, tachycardic, cachexic, with patchy and nodular shadowing of right upper zone on CXR. - Commenced on RHZE, pyridoxine, prophylactic CTX. | | After 2 weeks of TB treatment, loss of appetite, night sweats and fever had resolved and HAART was initiated. | - Although continuing to take HAART, did not attend for medical review until 6 weeks after initiation. - Has deteriorated with vomiting, loss of appetite, unable to cook for herself; has lost weight, redeveloped high fever and become jaundiced with tender hepatomegaly. CXR appearances also worsened with extensive consolidation of right upper lobe. USS showed abdominal lymph nodes, large liver with abnormal texture. Liver biopsy performed. | After liver biopsy results a diagnosis of probable TB-IRIS was made. HAART and TB therapy were continued but steroids were not given as a confirmed culture result was not available at that time.  Over the next 4 weeks all symptoms resolved, became apyrexial, jaundice resolved & gained weight. Induced sputum cultures from time of TB diagnosis confirmed fully sensitive M-tb. |
|  | LFTs at time of initial presentation: |  | LFTs at time of presentation with jaundice: | LFTs 3 weeks after presentation with jaundice: |
| Alb | 23 |  | 19 | 25 |
| Bil total | 3 |  | 42 | 17 |
| ALT | 8 |  | 42 | 25 |
| ALP | 91 |  | 174 | 101 |
| GGT | 30 |  | 105 | 79 |
| **Patient 4: 41 year old male; CD4 123 cells/µL** | | | | |
| - 3 months of dry cough; 3 weeks chest pain, fever. Not improved on broad spectrum antibiotics he had received as an inpatient. - On examination: pyrexial, tachycardia, grade III ulcers on pressure areas of back, 1.5cm lymph node in left supraclavicular area and tender abdomen in right upper quadrant. - CXR showed widespread nodular shadowing probable mediastinallymphadenopathy. - Commenced on RHZE, pyridoxine, prophylactic CTX. | | After 4 weeks TB treatment reported symptomatic improvement; temperature and heart rate normal, ulcers resolved, lymph node persisted at 1.5cm and abdomen was non-tender. | - After 2 weeks HAART he had deteriorated symptomatically and recurrent tachycardia & fever. Found to have jaundice and an enlarged tender liver. The supraclavicular LN remained 1.5cm but CXR showed dramatically worse lymphadenopathy and nodular shadowing. USS abdomen identified new epigastric lymphadenopathy and hepato-splenomegaly with normal biliary tree. | A presumptive diagnosis of TB-IRIS was made, prescribed prednisolone 40mg od po for 2 weeks followed by a weaning dose schedule, while HAART and RHZE continued. This resulted in resolution of fever, tachycardia, jaundice and hepatomegaly over 2 weeks. Induced sputum culture confirmed fully sensitive M-tb. |
|  | LFTs at time of initial presentation: | LFTs at time HAART initiation: | LFTs at time of presentation with jaundice: | LFTs 2 weeks after presentation with jaundice (after prednisolone): |
| Alb | 29 | 21 | 22 | 22 |
| Bil total | 8 | 9 | 40 | 23 |
| ALT | 105 | 47 | 293 | 122 |
| ALP | 442 | 737 | 1009 | 768 |
| GGT | 277 | 415 | 824 | 829 |

TB=Tuberculosis; CXR=Chest radiograph; USS=Ultrasound Scan; RHZE=Rifampicin, Isoniazid, Pyrazinamide, Ethambutolie quadruple TB therapy as per the South African national guidelines, RHZES indicates addition of streptomycin for ‘retreatment’ TB cases; HAART=Highly Active Antiretroviral Therapy which in all cases was Efavirenz, Lamivudine and Tenofovir prescribed as per South African national guidelines; CTX=Co-trimoxazole; LFTs=Liver function tests. CMV=Cytomegalovirus; AFB=Acid Fast Bacilli; M.tb= Mycobacterium tuberculosis. Alb = albumin in g/L; Bil total = total bilirubin µmol/L; ALT = alanine aminotransterase iµ/L; ALP = alkaline phosphatase iµ/L; GGT = Gamma-glutamyltranspeptidase iµ/L.

**Table S2: Application of Meintjes et al Paradoxical TB Case Definition to cases**

| **Patient** | **1** | **2** | **3** | **4** | **5** |
| --- | --- | --- | --- | --- | --- |
| **Antecedent requirements?** | | | | | |
| **MTB positive culture** | Yes | Yes | Yes | Yes | No |
| **Definite MTB in liver (AFB or PCR +)** | Yes, both | Yes, PCR + | Yes, PCR + | No, inadequate sample | No |
| **Initial response to TB Rx** | Yes | Yes | Yes | Yes | Yes |
| **Clinical criteria?** | | | | | |
| **Major criteria** | Yes, worsening CXR | yes, new lymphadenopathy and reaccumulated effusion | Yes, worsened CXR | Yes worse CXR and new lymphadenopathy | Yes, new effusion |
| **Minor criteria** | Yes, worsening constitutional s | Yes, worsening constitutional symptoms | Yes, worsening constitutional symptoms | Yes, worsening constitutional symptoms | Yes, worsening constitutional symptoms |
| **Alternate explanations excluded?** | | | | | |
| **Resistance** | Excluded | DST not available | Excluded | Excluded | No positive culture |
| **Poor adherence** | Excluded | Excluded | Excluded | Excluded | Excluded |
| **Other opportunistic infection or neoplasm** | No evidence | No evidence | No evidence | No evidence | Diarrhoea unexplained |
| **Drug reaction** | Possible, but inadequate to explain all findings | Possible, but inadequate to explain all findings | Possible, but inadequate to explain all findings | Possible, but inadequate to explain all findings | Probable |
| **Other** |  | Multiple aetiologies found | Multiple aetiologies found |  |  |

Key: AFB: acid fast bacilli; CXR: chest radiograph; DST: Direct sensitivity testing; MTB: Mycobacterium tuberculosis; PCR: polymerase chain reaction; Rx: treatment; TB: tuberculosis; +: positive.
